# Evaluation of the Effects of Oral N-Acetylcysteine and a Placebo in Paraclinical and Oxidative Stress Parameters of Patients with Chronic Hepatitis B

**Published:** 2010-06-01

**Authors:** Majid Shohrati, Fatemeh Dermanaki, Fatemeh Babaei, Seyed Moayed Alavian

**Affiliations:** 1 Research Center of Chemical Injuries, Baqiyatallah University of Medical Sciences, Tehran, I.R. Iran; 2 Faculty of Pharmacy, Islamic Azad University-Pharmaceutical Science Branch, Tehran, I.R. Iran; 3 Department of Biochemistry, Payam-e-Noor University, Tehran, I.R. Iran; 4 Research Center for Gastroenterology and Liver Disease, Baqiyatallah University of Medical Sciences, Tehran, I.R. Iran

**Keywords:** Hepatitis B, N-Acetylcysteine, Oxidative Stress

## Abstract

**Background and Aims:**

The treatment of chronic hepatitis B (CHB) is a challenging problem today, and previous study has shown that oxidative stress causes the collective pathophysiological conditions of many hepatopathies, so other new therapeutic approaches are needed. Hence, in this study the paraclinical and oxidative stress parameters of the efficacy of N-acetyl cysteine (NAC) as an antioxidant in the treatment of CHB have been evaluated.

**Methods:**

In this double-blind placebo-controlled clinical trial study, 43 patients with CHB were enrolled in 2008 in Tehran, Iran. The patients were randomly assigned to receive either 1200 mg/day NAC or a placebo for 45 days. Paraclinical tests and oxidative stress parameters were measured on experimental day 0 and on day 45.

**Results:**

Liver function tests, i.e. alanine aminotransferase (ALT), aspartate aminotransferase (AST) and alkaline phosphatase (ALP) levels were not significantly different in the NAC group and in the placebo group. A reduction in catalase (CAT) activity and an increase in glutathione concentration were statistically significant in the NAC group (P < 0.05).

**Conclusions:**

According to our results, oral NAC is not an effective adjuvant treatment for patients with CHB, but further research with a larger population is needed for the evaluation of the effectiveness of NAC in these patients.

## Introduction

It is estimated that over 2 billion people have been exposed to hepatitis B virus (HBV) worldwide [1]. About 350 million of the world’s population have been infected with HBV [2]; for the year 2000 the model estimated that 620000 patients died from HBV-related causes in the world: 6% from acute hepatitis B (AHB), and 94% from chronic infection related hepatocellular carcinoma and cirrhosis [3].

HBV infection is an important public health problem in the Middle East [4]. Previous studies showed that the prevalence of hepatitis B in Iran ranges between 1.3 and 8.69 percent in different regions [5]. Alavian et al. reported that about 1.5 million people in Iran are living with HBV infection, and it is assumed that 15% to 40% of them are at risk for developing cirrhosis and/or hepatocellular carcinoma (HCC), without intervention [6].

The goal of treatment for patients with chronic hepatitis B (CHB) infection is to prevent the progression of liver disease to cirrhosis and hepatocellular carcinoma [7]. The first aim of antiviral therapy is the reduction of serum HBV DNA to the lowest possible levels [8] and to improve liver necroinflammation. This is achieved by the normalization of serum alanine aminotransferase (ALT) levels, before liver fibrosis progresses to an irreversible stage [9].One useful treatment for chronic hepatitis is Interferon-alpha (IFN-a), but resistance to this drug occurs frequently [10]. Also, IFN-α therapy alone, for Chronic Hepatitis C (CHC) is not satisfactory, because more than two third of treated patients will not have a sustained response; thus, it would be desirable to add some new effective drugs for use in combination therapy to improve the response rate.[11].

Also, many side effects such as flu-like symptoms, bone marrow suppression and pancreatitis may be seen during IFN-α or lamivudine therapy [12][3].

Oxidative stress results from the extreme production of oxidant species in an extracellular environment. A major disturbance in the balance of prooxidants and antioxidants can lead to cellular damage [14]. Some researchers have investigated the relationship between oxidative stress and antioxidants in hepatitis virus infections and have shown that alternation in redox balance characterizes several viral infections and the development of virus-induced diseases [15]. In many liver diseases, inflammation (a source of tumor necrosis factor and exogenous reactive oxygen species [ROS]) caused by viral infection, increases the speed of the killing of liver cells, which may be sensitized by redox disturbance (ROS and/or thiol-disulfide) in some sub-cellular compartments [16]. When oxidative stress occurs in these patients, free radicals are controlled by many antioxidants, such as glutathione (GSH). In addition, experimental and clinical data indicate that the production of GSH is reduced in patients with chronic viral diseases, like human immunodeficiency virus (HIV) infection and CHC [11][17]. Some researchers have suggested that NAC, a precursor of GSH, has an anti-HBV DNA effect and could inhibit the replication of HBV in vitro [17][18].

Many studies have considered combination therapy with NAC plus IFN-α or lamivudine, for the treatment of CHB and CHC, but their results are controversial [10][19][20]; so these findings prompted us to carry out a randomized, double-blind, placebo-controlled study to assess the beneficial effects of combination oral NAC therapy in paraclinical tests and oxidative stress parameters in CHB patients.

## Materials and Methods

In this study, 43 CHB patients from the Tehran Hepatitis Center in Tehran, Iran were consecutively chosen in 2008 for this randomized double-blind placebo-controlled trial. They had been receiving CHB drugs including lamivudine, pegylated interferon, and adefovir. The patients were randomly assigned to two groups: the NAC group receiving 1200 mg/day (Fluimucil, the Zambon Co.) for 45 days, and a matching placebo group. Inclusion criteria were as follows: a documented diagnosis of Hepatitis B, non-participation in any other study, and the non-use of any kind of drugs that would interact with those used in this study. Exclusion criteria were: the occurrence of any severe side effects of NAC, the use of any kind of antioxidant drugs and the taking of less than 80% of the medication. Tablets of both drug and placebo were prepared by the Zambon Company. Patients and physicians were blind to the kind of drug or placebo and so was the other research team. Patients enrolled in the study were from a clinic in Tehran, Iran. Levels of biochemical parameters, e.g. total bilirubin (TBil) ,direct bilirubin (DBil), alanine aminotransferase (ALT), aspartate aminotransferase (AST), alkaline phosphatase (ALP), prothrombine time (PT), GSH, catalase (CAT) and superoxide dismutase (SOD) were monitored in both the NAC and the placebo group at experimental day 0 and day 45.

The study was approved by the ethical committee of Baqiyatallah University of Medical Sciences. Written informed consent was obtained from all patients before the study was carried out.

Statistical analysis was performed by using SPSS software (Statistical Procedures for the Social Sciences (version 14.0), Chicago, Illinois, USA). Independent sample, paired sample T and Mann-Whitney U tests were performed. P values of less than 0.05 were considered significant.

Venous blood samples were collected in vacuum tubes containing EDTA as the anticoagulant. Plasma samples were separated from blood cells and stored at -80°C and were used for measuring GSH levels, CAT and SOD activities.

GSH levels were determined using a modified version of the Tietze method [21]. Cellular protein was precipitated by the addition of 5% sulfosalisylic acid and removed by centrifugation at 3000×g for 15 min. GSH was assayed in the supernatant as follows: to 100 µl of the protein-free cell lysate supernatant were added 800 µl of 0.3 mM Na2HPO4 and 100µl of 0.04% 5, 5-dithiobis 2-nitrobenzoic acid (DTNB) in 0.1% sodium citrate. The absorbance of the solution was monitored at 412 nm for 5 min using the Beckman UV spectrophotometer. The value for each sample was read from the standard curve and was expressed in nmol/ml.

CAT activity was determined following the decomposition of H2O2 according to the Cohen method [22].The reaction was started by mixing H2O2 (6 mM) to the plasma and phosphate buffer (50 mM at pH 7.0), and stopped by adding H2SO4 (6 N) after 3 minutes. Then KMnO4 (0.01 N) was added and absorbance measured by means of the spectrophotometer at 480 nm. The activity of the enzyme was expressed as units per liter.

SOD activity was measured by the inhibition of NADPH oxidation [23]. For assay, TDB buffer (triethanolamine – diethanolamine (0.1M each) – 1.38% HCl buffer at pH 7.4) was added to cuvettes, followed by 0.27 mM NADH, 5 mM EDTA, 2.5 mM MnCl2 and 0.1 ml of the sample. 2-mercaptoethanol (3.75 mM) was added to the mixture and the decrease in absorbance was monitored after 5 minutes. One unit of activity is defined as the amount of enzyme that inhibits the oxidation of NADH by 50% at 25oC.

## Results

The mean age of patients was not significantly different between the NAC group and the placebo group. Out of the 43 patients who enrolled in this study, 4 patients, 2 in the NAC group and 2 in the placebo group had their participation in the study cancelledand one patient in the NAC group was discontinued because of vomiting. Of the remaining 38 participants, in the NAC group, 14 patients (77%) were male, and 18 patients (90%) in the placebo group. The Mean ± standard deviation (SD) age of the participants in the NAC and the placebo group was 44.33±13.40, 43.9±13.34, respectively. NAC was tolerated without any serious side effects.

ALT, AST, ALP and DBil concentrations significantly decreased after administration of NAC (P = 0.004, P = 0.024, P = 0.014 and P = 0.01), respectively. These parameters had no significant change in the placebo group, except for the AST. Also, these parameters were not significantly different in the NAC and the placebo group (P > 0.05). Other biochemical parameter changes are shown in (Table 1).

Changes in SOD activity in both the NAC and the placebo group were not significantly different at day 45 in comparison to the baseline. Also, there was no significant difference between the NAC and the placebo group (P > 0.05)Table1.

The mean CAT activity in the NAC group before (14.76 ± 2.45 U/L) and after (8.53±1.43 U/L) the study showed a significant decrease (P = 0.02). There was no significant difference in CAT activity in the placebo group, both before and after the study (P> 0.05). Also, there was no significant difference in CAT activity in the NAC and the placebo group (P > 0.05) (Table1).

There was a significant increase in GSH concentration in the NAC group, before (7.5 ± 1.27 nmol/ml), and after (11.76±2.01) the study (P = 0.001).There was no significant difference in GSH concentration in the placebo group, both before and after the study (P > 0.05). There was no significant difference in GSH concentration in the NAC and the placebo group. (P > 0.05) (Table1).

**Table1:** Oxidative stress parameters and biochemical characteristics at baseline and 45 days after treatment with NAC and Placebo groups.

**Parameters**		**NAC (n=18)**	**P-value**	**Placebo (n=20)**	**P-value**
Age(Mean± SD) year		44.33±13.40	-	43.9±13.34	-
Sex	Male/Female	14/4	-	18/2	-
ALT(U/L)	Base line	76.22 ± 32.7	0.004	91.95± 48.8	P > 0.05
	After 45 days	51.00±22.3		70.50± 60.6	
AST(U/L)	Base line	57.83±19.4	0.024	67.60 ± 32.7	0.018
	After 45 days	48.06±19.8		48.90± 33.4	
ALP(U/L)	Base line	237.87±141.7	0.014	211.90 ±86.2	P > 0.05
	After 45 days	134.80±34.3		187.90 ±78.6	
DBil(mg/dl)	Base line	0.33 ± 0.23	0.01	0.22 ± 0.08	P > 0.05
	After 45 days	0.28 ± 0.19		0.21 ±0.08	
TBil(mg/dl)	Base line	1.10 ± 0.69	P > 0.05	0.74 ±0.17	P > 0.05
	After 45 days	0.99 ± 0.38		0.81±0.25	
PT(sec)	Base line	13.48 ± 1.67	P > 0.05	13.59 ± 1.12	P > 0.05
	After 45 days	12.55± 1.16		13.14±0.8	
SOD(U/L)	Base line	3.39 ± 0.40	P > 0.05	3.87 ± 0.38	P > 0.05
	After 45 days	3.29±0.42		3.84±0.39	
CAT(U/L)	Base line	14.76 ± 2.45	0.02	12.86 ±1.93	P > 0.05
	After 45 days	8.53±1.43		10.66±1.75	
GSH(nmol/mL)	Base line	7.5 ± 1.27	0.001	8.48 ± 1.74	P > 0.05
	After 45 days	11.76±2.01		9.96±1.44	

## Discussion

Recently, combination therapy with vitamin E, or NAC and IFN-α, has been considered an attractive option for hepatitis patients [10]. It is assumed that during HCV infection, vitamin E and NAC counteract oxidative stress in the hepatic microenvironment caused by activated Kupffer cells and infiltrating mononuclear cells [24]. Weiss et al. reported that NAC restrains HBV replication in vitro, but that the mechanism of NAC was independent of the intracellular level of ROS. They also showed that NAC decreased viral DNA in the tissue culture supernatant in vitro at least 50-fold within 48 hours. This decrease in viral DNA resulted in the disturbance of the virus assembly [18].

Our results showed that TBil and PT decreased in the NAC group, but this reduction was not significant when compared with the placebo group. Gunduz et al. reported that 600 mg/ day oral administration of NAC has no effect on a decrease in TBiL and an increase in the activity of prothrombine (PTA) [19]. Another study showed that the injection of NAC can decrease the level of serum TBil and increase the PTA [20]. In addition, Wang and colleagues assessed that both NAC and GSH injection decreased TBil of serum and increased PTA in CHB patients, but that NAC was more efficient in decreasing TBil than GSH [25].

Our study showed a significant reduction in ALT in the NAC group and no significant reduction in the placebo group; and a significant reduction in AST in the NAC group and in the placebo group as well. Our data are in line with those of another study by Gunduz et al. [19]. Moreover Grant et al. reported serum ALT levels did not differ significantly between co-therapy with IFN-α plus NAC, and IFN-α therapy alone [26]. In agreement with our results, Bernhard et al. reported that there was no statistically significant difference in ALT and AST between a dose of 1800 mg/day of NAC and a placebo.[11]. However, another study could not confirm our observation; the authors reported that the serum ALT levels of 41% of patients were normalized after 6 months of IFN-α plus NAC [27].

Similarly to our findings, Look et al. reported no significant advantage of an antioxidant plus IFN in combination therapy, in terms of response, after 6 months, compared to IFN monotherapy [24]. Also, Ideo et al. reported that a combination of IFN-α with NAC and vitamin E was not effective in biochemical responses rates and clearance of the virus from the serum of CHC patients was not observed [28]. Our data are also in line with those of another study by Bernhard and colleagues, who did not find beneficial response rates when IFN was combined with NAC, in comparison with IFN-α alone [11].

Our results showed that 1200 mg/day oral administration of NAC for 45 days is tolerated by patients. We did not observe any serious adverse effects during the 45 days, and these findings are in line with those of the study by Weidenbach et al., who reported that CHB patients could tolerate intravenous NAC without any side effects for 28 days, but NAC therapy could not have changed HBV-DNA levels significantly [29].

Our results demonstrated that administration of NAC reduced CAT activity and caused a rise in GSH concentration in the NAC group, but no significant reduction of SOD activity was observed in this group.

SOD catalyzes in order to scavenge excess superoxide anions and convert them to H2O2 and other enzymes like CAT and glutathione peroxidase scavenge H2O2 and convert it to water [30]. Investigation has shown that NAC is able to clean the cell by scavenging the superoxide anion radicals; therefore a reduction of SOD and CAT activity seems logical [31]. Researchers have reported that erythrocyte SOD activity in children with CHC and CHB significantly decreased in comparison to a healthy control group [32]; in addition, another study showed that erythrocyte SOD activity increased in acute hepatitis C patients, but decreased in CHC patients, and this decrease was reversed when the patients were treated with interferon [33]. In the Liu et al. study, liver SOD activity showed a remarkable increase, when mice were treated with NAC in different doses, suggesting that NAC induces SOD activity [34]. Ozaras et al. showed that NAC elevated SOD activity in the liver of rats given alcohol treatment [35].

Goth et al. reported serum CAT activity in hepatitis patients increased [36], and Bay et al., in 2002, reported CAT activity in CHC patients significantly increased but decreased in CHC patients with interferon therapy. [32]. Other research also showed that erythrocyte CAT activity in children with CHB and CHC significantly decreased in comparison with a healthy control group [33].

One of the key events in hepatocellular damage is a decrease in cellular glutathione levels, particularly in the mitochondrial ‘slow-turnover’- GSH pool. GSH is one of the key constituents of the host’s antioxidative capacity. It contributes to effective immune surveillance and to xenobiotic metabolism. Since GSH itself is not transported over bio-membranes and cannot be substituted directly, its abundance might be possible by providing precursors such as NAC or GSH esters [27]. The results of this study showed that GSH concentration increased in the NAC group but this increase was not statistically significant between the NAC and the placebo group. Our results are in accordance with the previous study. Bernhard and colleagues showed that 1800 mg/day of NAC therapy in CHC patients could increase the level of GSH after 12 weeks, but this improvement was not significant statistically with the placebo group [11]. Look et al. found that GSH concentration was elevated after administration of NAC in a dose of 1800 mg/day to chronic hepatitis C patients [24]; Arfsten et al. reported GSH concentration increased 20% in the skin and 50% in the liver after 1200 mg/kg of NAC was administered, whereas lung and kidney GSH were unaffected in rats [37]. Further, another study showed that neither interferon nor NAC significantly affected GSH concentration in patients [38].The author of this article reported in another paper the efficacy of NAC in doses of 1200 mg and 1800 mg in the clinical improvement of chronic lung disease caused by sulfur mustard; and also reported on the parameters of oxidative stress in these patients. The dose of 1200 mg in this study is based on the previous study [38][39][40].

## Conclusions

Supplementation with NAC did not have any effect on paraclinical tests and the plasma concentration of SOD, CAT and glutathione in our patients. The administration of NAC in HBV patients is based on the hypothesis that the supplementation of a prodrug of cysteine, the rate-limiting amino acid in glutathione synthesis, will result in an increased concentration of antioxidant capacity, so no definite conclusions can be drawn from the present study: 45 days is not enough to draw any conclusion, and more time may be needed.
